# Reply to Byker Shanks et al. Measurement of Fruit and Vegetable Intake Incorporating a Diversity, Equity, and Inclusion Lens. Comment on “Di Noia, J.; Gellermann, W. Use of the Spectroscopy-Based Veggie Meter^®^ to Objectively Assess Fruit and Vegetable Intake in Low-Income Adults. *Nutrients* 2021, *13*, 2270”

**DOI:** 10.3390/nu14040811

**Published:** 2022-02-15

**Authors:** Jennifer Di Noia, Werner Gellermann

**Affiliations:** 1 Department of Sociology, William Paterson University, Wayne, NJ 07470, USA; 2 Longevity Link Corporation, Salt Lake City, UT 84108, USA; werner@longevitylinkcorporation.com

We thank Byker Shanks et al. [1] for their interest in our work. In their commentary, the authors describe the Veggie Meter (VM) as using resonance Raman spectrometry. There are currently two optical methods for assessing skin carotenoid status for nutritional studies, as follows: resonance Raman spectroscopy (RRS) and pressure-mediated reflection spectroscopy (RS) [2]. The VM, which is commercially available, uses the latter method. The authors further reference a review of research examining correlations between RRS-assessed skin carotenoids and plasma/serum carotenoids, noting that the validity of this method has been mainly examined among white populations [3]. RS measurement of skin carotenoids is a relatively recent development [2]; nevertheless, there is a growing body of research demonstrating the validity of RS in cohorts of different age groups and ethnicities in community and clinical settings [4,5,6,7,8,9]. VM scores have also been reported in many different groups as summarized in our article [10]. These findings highlight ongoing efforts to aid understanding of the utility of the VM for assessing fruit and vegetable consumption in diverse populations.

We fully agree that measures of diversity, equity, and inclusion are important for understanding such influences on fruit and vegetable consumption as individual differences in the variety and types of fruits and vegetables consumed, fruit and vegetable access barriers, and sociodemographic variables and languages with which people identify. Assessing these variables will further refine our understanding of dietary patterns and influencing factors in diverse groups.
